# Predictors of secondary *streptococcus pneumoniae* and/or *haemophilus influenzae* infection in children hospitalised with RSV, and development of a clinical prediction model to inform antibiotic decisions: a retrospective cohort study based on coloniser–pathogen distinction

**DOI:** 10.3389/fped.2026.1915356

**Published:** 2026-10-28

**Authors:** Jingwen Ni, Xiaoqiao Zhang, Junyu Dong, Mengxin Zhao, Feiyan Chen, Kenan Fang

**Affiliations:** Pediatric Intensive Care Unit, Luoyang Maternal and Child Health Hospital, Luoyang, China

**Keywords:** children, *H. influenzae*, predictors, RSV, *S. pneumoniae*

## Abstract

**Background:**

Respiratory syncytial virus (RSV) is the leading cause of acute respiratory tract infection (ARTI) in infants and young children. Co-infection with *Streptococcus pneumoniae* and/or *Haemophilus influenzae* substantially increases disease severity. However, the detection rates of these bacteria in the nasopharynx of young children vary considerably, which can lead to overestimation of bacterial infection and inappropriate antibiotic use. This study aims to develop a nomogram for predicting secondary *S. pneumoniae* and/or *H. influenzae* infection in children hospitalised with RSV, based on a rigorous distinction between colonisation and true pathogenicity.

**Methods:**

We conducted a retrospective, single-centre study of children hospitalised with ARTI due to RSV between January 2023 and October 2025. According to predefined criteria for bacterial infection, 1,573 patients were identified and randomly assigned in a 7:3 ratio to a training set (*n* = 1,104) and an internal validation set (*n* = 469). Variable selection was performed using least absolute shrinkage and selection operator (LASSO) regression with ten-fold cross-validation. A nomogram was then constructed using multivariable logistic regression. Model performance was assessed by the area under the receiver operating characteristic curve (AUC), calibration curves, and decision curve analysis.

**Result:**

Multivariable logistic regression identified lung consolidation, RSV-CLASS, WBC, NLR, SF, and IL-6 as independent predictors. The nomogram achieved an AUC of 0.872 (95% CI: 0.841–0.898) in the training set and 0.829 (95% CI: 0.780–0.877) in the internal validation set, with an optimism-corrected C-statistic of 0.864. Calibration showed miscalibration in the internal validation set (slope 0.762, intercept −0.350), indicating a tendency to overestimate risk, and decision curve analysis indicated net benefit at threshold probabilities of 0.2–0.6.

**Conclusion:**

Based on a rigorous distinction between colonising and pathogenic bacteria, this study constructed a nomogram integrating six variables that combine clinical, laboratory, and imaging findings. This nomogram offers clinicians a reference when considering antibiotics for children hospitalised with RSV and secondary *S. pneumoniae* or *H. influenzae* infection, but its current utility is primarily as a rule-out tool given the low PPV; it should not be used alone to guide antibiotic initiation. External validation is required before broader clinical implementation.

## 1 Background

Respiratory syncytial virus (RSV) is the leading cause of acute respiratory tract infection (ARTI) in infants and young children worldwide. In 2015, an estimated 33.1 million episodes of RSV-associated acute lower respiratory tract infection occurred, resulting in 3.2 million hospitalisations and as many as 118 200 deaths among young children (1). However, the global disease burden of RSV among children under 5 years of age continues to increase. Recent studies indicate that one in 50 deaths in this age group is associated with RSV, and among children aged 28 days to 6 months, one in 28 deaths is attributable to RSV (2). In early 2026, the latest data from the Chinese Center for Disease Control and Prevention showed that the positive detection rate of RSV among hospitalised patients with severe acute respiratory infection had risen to 13.3%, ranking first among all respiratory pathogens. In parallel, substantial progress has been made in the development of RSV prophylactic products, such as nirsevimab and maternal vaccination during pregnancy. In paediatric patients, nirsevimab has been shown to significantly reduce RSV-related hospitalisation rates, bronchiolitis severity, and paediatric intensive care unit (PICU) admissions (3).

However, the substantially greater clinical burden associated with RSV and bacterial co-infection remains a major concern. *Streptococcus pneumoniae* and *Haemophilus influenzae* are the two most frequently detected bacteria in this context, and their presence leads to more severe clinical symptoms, a higher likelihood of PICU admission, and an increased risk of death in affected children (4–8).

Globally, the prevalence of bacterial co-detection in children with RSV infection varies substantially, ranging from 15.7% to 39%. The core driver of this variation is the inability of routine diagnostic methods to distinguish between upper respiratory tract bacterial colonisation and true secondary infection (7, 9–12). Children have a high nasopharyngeal colonisation rate of *S. pneumoniae* and *H. influenzae*, and the bacterial detection rate can still exceed 40% even in mild RSV cases. This leads clinicians to misattribute colonisation to infection and to prescribe empirical antibiotics (13). Most existing prediction models use a positive nucleic acid test for bacteria as the outcome, without distinguishing between colonising bacteria and true pathogens. This may lead to overestimation of infection risk and inappropriate antibiotic decision-making (14, 15). Therefore, we developed and internally validated a nomogram to predict secondary *S. pneumoniae* or *H. influenzae* infection in children with RSV. The model distinguishes colonisers from true pathogens, and is intended to support more rational antibiotic use.

## 2 Methods

### 2.1 Study design and population

This retrospective, single-centre study included children with RSV infection who were admitted to Luoyang Maternal and Child Health Hospital (Henan, China) between 1 January 2023 and 31 October 2025.

Inclusion criteria: (1) Age 29 days to 18 years. (2) Etiological confirmation of RSV infection. (3) Definite diagnosis of ARTI (16). (4) Complete hospital records available.

Exclusion criteria: (1) Children who acquired RSV infection due to nosocomial infection during hospitalisation. (2) Children without acute respiratory tract infection.

#### 2.1.1 Sampling method

##### 2.1.1.1 Sample collection timing

All samples were collected as early as possible during the acute phase of hospitalisation, specifically within 24 h of admission (or within 24 h of intubation for mechanically ventilated children).

##### 2.1.1.2 Standard operating procedure

2.1 For children not receiving invasive mechanical ventilation, sputum specimens were collected by nasopharyngeal or oropharyngeal aspiration under sterile conditions. When sputum aspiration was not feasible, nasopharyngeal swabs were collected using sterile synthetic fibre swabs.

Sputum culture: When sputum was obtained, it was inoculated onto Columbia blood agar plates and incubated at 37 °C for 24 h.

Nasopharyngeal swab RT-PCR: When sputum was not available, nasopharyngeal swabs were tested by standard reverse transcription polymerase chain reaction (RT-PCR), which targeted a panel of common respiratory bacterial pathogens, including *Streptococcus pneumoniae*, *Haemophilus influenzae*, *Staphylococcus aureus*, *Klebsiella pneumoniae*, *Legionella pneumophila*, and *Pseudomonas aeruginosa*.

2.2 For intubated children receiving invasive mechanical ventilation, endotracheal secretions were aspirated using a suction device.

Metagenomic next-generation sequencing (mNGS): When financially feasible and with guardian consent, mNGS was performed on the aspirate, including nucleic acid extraction, reverse transcription (for RNA), library preparation, high-throughput sequencing, and bioinformatics analysis for species identification.

Sputum culture (alternative): When guardians declined mNGS due to cost, deep endotracheal sputum specimens were processed using the same culture method described in section 2.1.

2.3 For children who underwent fibreoptic bronchoscopy, bronchoalveolar lavage fluid (BALF) specimens were prioritized and tested either by mNGS or by sputum culture, following the same protocols described above, with guardian consent obtained beforehand.

### 2.2 Definition of study variables

At our institution, all children hospitalised with RSV infection routinely receive a standard admission panel that includes complete blood count, C-reactive protein, serum ferritin, and IL-6. These tests are part of the automatic order set for every admitted patient, not ordered selectively based on clinical suspicion. Thus, in our cohort, testing for these markers was broadly uniform and not biased toward more severely ill children.

Prematurity: Defined as a gestational age between 28 weeks and 36 weeks and 6 days (i.e., <37 weeks).

Atopic disorders: History of wheeze, eczema, or allergies (17).

RSV-CLASS: This scoring system includes four components: cough, tachypnea, rales, and wheezing. Each component is scored 1 point, and the final total score is calculated based on the clinical symptoms of the child (18).

NLR: Neutrophil-to-lymphocyte ratio (19).

PLR: Platelet-to-lymphocyte ratio (19).

Chest x-ray interpretation: Chest radiographs were obtained as part of routine clinical care for children with suspected pneumonia or severe respiratory illness. All images were interpreted by attending radiologists at our institution at the time of clinical presentation, and the radiology reports were subsequently reviewed for data extraction. Radiological findings, including the presence or absence of lung consolidation, pleural effusion, and pneumothorax, were recorded from the original reports.

In the absence of a universally accepted gold standard for diagnosing bacterial co-infection in children with RSV infection, we adopted a composite clinical classification to define secondary bacterial infection. In this study, secondary bacterial infection in children hospitalised with RSV was defined as meeting all three of the following criteria: (1) bacterial detection from nasopharyngeal swab, bronchoalveolar lavage fluid, or endotracheal aspirate. (2) clinical manifestations of acute infection (e.g., peak fever ≥ 39 °C with fever duration > 72 h, tachypnoea, lower chest wall indrawing, or lethargy) (13). (3) Laboratory inflammatory markers (procalcitonin > 0.25 ng/mL or C-reactive protein >40 mg/L) (13, 16). Children with bacterial detection but without corresponding clinical manifestations or inflammatory/imaging support and those with RSV alone and negative bacterial tests served as the control group. This composite definition avoids overestimation of infection risk based solely on bacterial detection.

### 2.3 Data collection

To avoid data errors, data collection for this study was jointly performed by six clinicians. Two of them completed the clinical medical records of all enrolled children by reviewing the electronic medical record system; two were responsible for retrieving and entering laboratory data; and the remaining two entered and cross-checked all the above data. All six clinicians participated in the determination and verification of secondary bacterial infection. Each patient was assigned a study identification number to ensure data privacy, and no identifiable personal data were collected.

### 2.4 Ethical statement and informed consent

This study was approved by the Ethics Committee of Luoyang Maternal and Child Health Hospital (approval number KY2022021401.0). Given the retrospective nature of the study and the use of anonymised patient data, the requirement for informed consent was waived in accordance with institutional and national ethical guidelines.

### 2.5 Statistical analysis

Data were processed and analyzed using SPSS 27.0 and R 4.5.1. Missing values were imputed using multiple imputation; however, cases with extensive missing radiological or laboratory data were excluded without imputation (see Supplementary Table S1). Categorical variables were expressed as counts (percentages) and compared using the chi-square test. For continuous variables, the Kolmogorov–Smirnov test was used to assess normality. Normally distributed data were presented as mean ± standard deviation (SD) and compared using the independent-samples t-test, while non-normally distributed data were presented as median (P25, P75) and compared using the Mann–Whitney *U* test. A *P*-value < 0.05 was considered statistically significant.

Patients were randomly divided into a training set (*n* = 1,104) and an internal validation set (*n* = 469) in a 7:3 ratio. In the training set, potential predictors were screened using LASSO regression with 10-fold cross-validation. The following candidate variables were entered into the LASSO regression: calendar month of onset, sex, prematurity, allergy history, wheezing history, cyanosis, fever, cough, wheezing, lung consolidation, pleural effusion, pneumothorax, breastfeeding, admission weight, age, RSV-CLASS, WBC, NLR, PLT, PLR, MPV, SF, IL-6, PT, APTT, D-dimer, and AT-III. All candidate variables were available within 24 h of admission. Variables reflecting in-hospital events or outcomes (e.g., PICU admission, respiratory support, oxygen treatment, mortality, and hospitalisation duration) were deliberately excluded from the candidate pool, as they would not be known at the time of prediction. Following LASSO selection, a multivariable logistic regression model was constructed. The discriminative ability of the model was evaluated using the area under the receiver operating characteristic curve (AUC). Internal validation was performed using the bootstrap method with 1,000 resamples to calculate the optimism-corrected C-statistic. The clinical applicability of the model was assessed by decision curve analysis (DCA), comparing the net benefit of the model with the “treat-all” and “treat-none” strategies within a reasonable range of threshold probabilities. A two-sided *P* < 0.05 was considered statistically significant.

Regarding sample size: the training set included 1,104 children, with 140 cases of positive outcome events. The final model contained six predictors, yielding an events-per-variable (EPV) ratio of 23.33, which meets the sample size requirement for model development.

This study was conducted and reported in accordance with the TRIPOD + AI statement. The completed checklist is provided in Supplementary Table S2.

## 3 Results

### 3.1 Recruitment of the enrolled children

A total of 2,141 children hospitalised with a positive RSV test were enrolled in this study, including 535 in 2023, 962 in 2024, and 644 from January to October 2025. After excluding five children with hospital-acquired RSV infection, 15 without acute respiratory symptoms, 27 who did not undergo bacterial testing, 18 who were discharged prematurely against medical advice before recovery, and 237 with incomplete data, 1,839 children passed the initial screening. Of the 1,839 children initially screened, 1,075 had no bacteria detected, while 764 tested positive for at least one bacterial pathogen—a bacterial detection rate of 41.5%. Among the 764 bacterially positive children, 498 were positive for *S. pneumoniae* and/or *H. influenzae*. We reviewed the medical records of these 498 children against pre-specified criteria for secondary bacterial infection and classified 199 as having true infection and the remaining 299 as colonisation. This gave a final analytic cohort of 1,573 children, consisting of 199 with bacterial co-infection and 1,374 without (the latter including 1,075 with no bacteria detected and 299 with colonising organisms), as shown in Figure 1.

**Figure 1:**
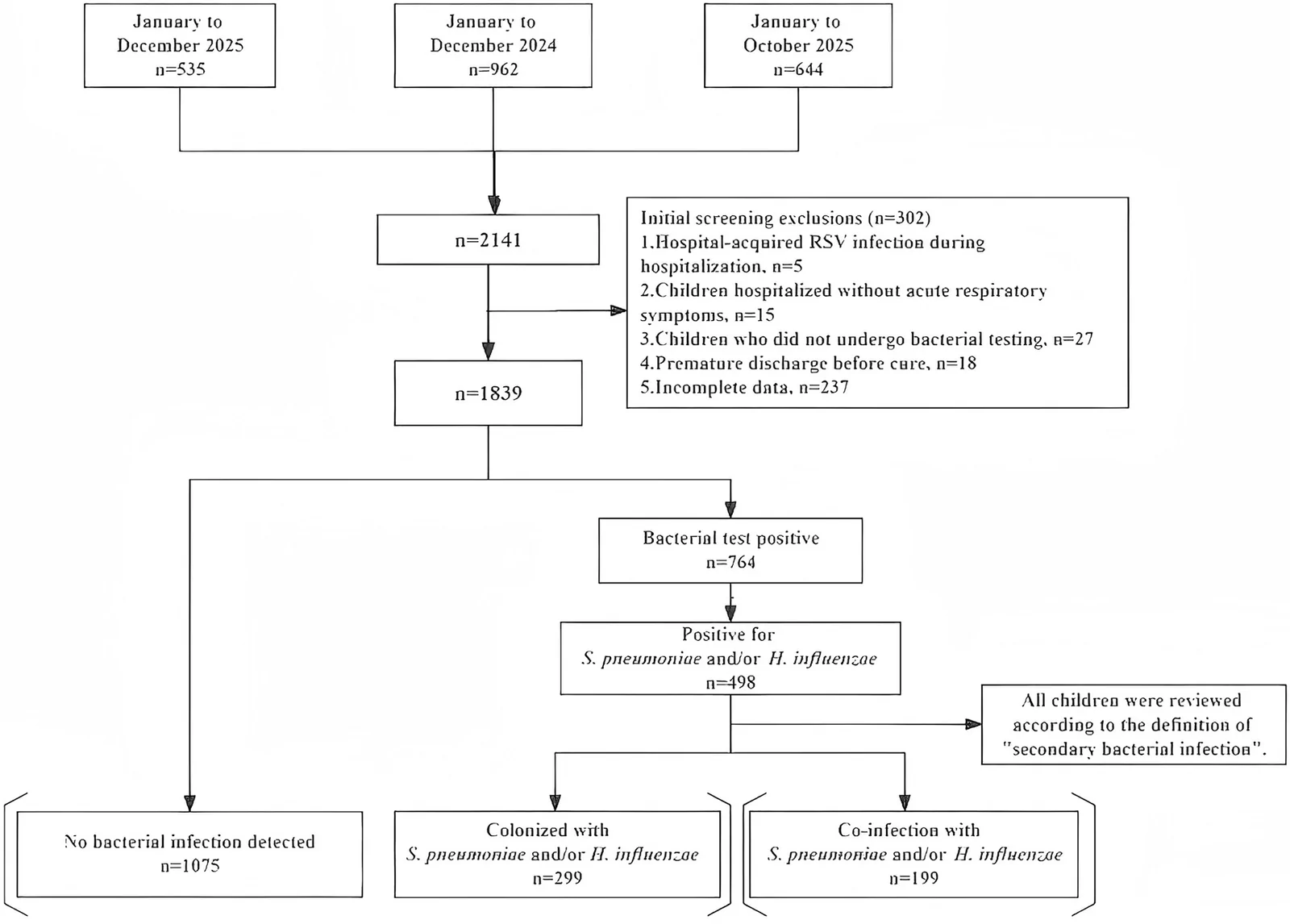
Flowchart of patient enrollment and recruitment.

In this cohort, bacterial detection was performed using sputum culture, nasopharyngeal swab RT-PCR, or mNGS, depending on clinical severity and family financial capacity. Specifically, mNGS was more frequently used in intubated children with severe illness, while culture and RT-PCR were more common in non-intubated or milder cases. This non-randomised allocation of detection methods may have introduced detection bias, which we have addressed in the Limitations.

### 3.2 Comparison of baseline characteristics between the non-bacterial infection group and the group with co-infection of *S. pneumoniae* and/or *H. influenzae*

The comparison revealed statistically significant differences between the two groups in terms of prematurity, cyanosis, fever, wheezing, dyspnea, lung consolidation, pleural fluid, pneumothorax, breastfeeding, oxygen treatment, admitted to the PICU, respiratory support, mortality, RSV-CLASS, WBC, NLR, CRP, PLR, PCT, SF, IL-6, PT, APTT, D-dimer, and hospitalization duration (all *P* < 0.05). No statistically significant differences were observed for the remaining variables (*P* > 0.05), as shown in Table 1.

**Table 1:** Comparison of baseline characteristics between the non-bacterial infection group and the group with co-infection of *S. pneumoniae* and/or *H. influenzae.*

Variables	No Bacterial Coinfection (*n* = 1,374)	Secondary bacterial infection with *S. pneumoniae* and/or *H. influenzae* (*n* = 199)	*χ*^2^/Z	*P*
Calendar month of onset, *n* (%)^1^			7.385	0.767
January, *n* (%)^1^	317 (23.07)	49 (24.62)		
February, *n* (%)^1^	241 (17.54)	37 (18.59)		
March, *n* (%)^1^	97 (7.06)	10 (5.03)		
April, *n* (%)^1^	316 (23)	49 (24.62)		
May, *n* (%)^1^	182 (13.25)	21 (10.55)		
June, *n* (%)^1^	26 (1.89)	6 (3.02)		
July, *n* (%)^1^	23 (1.67)	3 (1.51)		
August, *n* (%)^1^	16 (1.16)	5 (2.51)		
September, *n* (%)^1^	7 (0.51)	0 (0.00)		
October, *n* (%)^1^	11 (0.80)	1 (0.50)		
November, *n* (%)^1^	46 (3.35)	6 (3.02)		
December, *n* (%)^1^	92 (6.70)	12 (6.03)		
Male, *n* (%)^1^	824 (59.97)	123 (61.81)	0.245	0.621
Prematurity, *n* (%)^1^	58 (4.22)	17 (8.54)	7.149	0.007
Allergy history, *n* (%)^1^	132 (9.61)	18 (9.05)	0.064	0.801
Wheezing History, *n* (%)^1^	100 (7.28)	21 (10.55)	2.625	0.105
Cyanosis, *n* (%)^1^	11 (0.80)	15 (7.54)	48.535	<0.001
Fever, *n* (%)^1^	894 (65.07)	150 (75.38)	8.280	<0.001
Cough, *n* (%)^1^	1,354 (98.54)	197 (98.99)	0.256	0.613
Wheezing, *n* (%)^1^	305 (22.20)	67 (33.67)	12.666	<0.001
Dyspnea, *n* (%)^1^	51 (3.71)	43 (21.61)	99.082	<0.001
Lung consolidation, *n* (%)^1^	44 (3.20)	43 (21.61)	94.159	<0.001
Pleural Fluid, *n* (%)^1^	14 (1.02)	9 (4.52)	14.810	<0.001
Pneumothorax, *n* (%)^1^	0 (0.00)	1 (0.50)	6.909	0.009
Breastfeeding, *n* (%)^1^	778 (56.62)	92 (46.23)	7.594	0.006
Oxygen treatmen, *n* (%)t^1^	168 (12.23)	68 (34.17)	65.637	<0.001
Admitted to the PICU, *n* (%)^1^	63 (4.59)	47 (23.62)	96.815	<0.001
Respiratory support, *n* (%)^1^			183.895	<0.001
Invasive, *n* (%)	4 (0.29)	24 (12.06)		
Non-invasive, *n* (%)	4 (0.29)	10 (5.03)		
Mortality, *n* (%)^1^	1 (0.07)	2 (1.01)	7.936	0.005
Admission weight (kg), M (Q₁, Q₃)^2^	10.00 (8.00,14.00)	11.00 (8.00,14.00)	−0.302	0.763
Age (months), M (Q₁, Q₃)^2^	12.00 (5.61,31.00)	16.00 (6.43,33.00)	−1.209	0.227
RSV-CLASS, M (Q₁, Q₃)^2^	2.00 (2.00,3.00)	2.00 (2.00,3.00)	−5.141	<0.001
WBC (×10^9^/L), M (Q₁, Q₃)^2^	8.75 (6.47,11.08)	10.16 (7.52,13.72)	−5.452	<0.001
NLR, M (Q₁, Q₃)^2^	0.47 (0.25,0.95)	1.31 (0.71,2.71)	−12.590	<0.001
CRP (mg/L), M (Q₁, Q₃)^2^	1.54 (0.43,5.80)	13.29 (4.49,27.64)	−14.296	<0.001
PLT (×10^9^/L), M (Q₁, Q₃)^2^	354.50 (283.00,431.00)	363.00 (270.00,465.00)	−0.870	0.384
PLR, M (Q₁, Q₃)^2^	66.16 (43.62,103.16)	98.03 (64.76,151.95)	−7.588	<0.001
MPV (fL), M (Q₁, Q₃)^2^	9.00 (8.50,9.70)	9.20 (8.60,9.90)	−1.722	0.085
PCT (ng/mL), M (Q₁, Q₃)^2^	0.10 (0.06,0.13)	0.32 (0.16,0.60)	−16.023	<0.001
SF (ng/mL), M (Q₁, Q₃)^2^	59.74 (29.01,104.86)	99.44 (51.50,163.43)	−7.343	<0.001
IL-6 (pg/mL), M (Q₁, Q₃)^2^	6.99 (2.87,15.81)	40.12 (20.90,67.05)	−15.162	<0.001
PT (s), M (Q₁, Q₃)^2^	11.40 (10.70,12.43)	11.90 (11.30,12.70)	−5.137	<0.001
APTT (s), M (Q₁, Q₃)^2^	29.98 (26.79,32.93)	30.70 (27.99,35.10)	−2.346	0.019
D-Dimer (ug/mL), M (Q₁, Q₃)^2^	0.33 (0.22,0.45)	0.40 (0.29,0.54)	−5.255	<0.001
AT-III (%), M (Q₁, Q₃)^2^	103.70 (94.50,110.93)	103.40 (94.10,110.50)	−0.845	0.398
Hospitalization duration (d), M (Q₁, Q₃)^2^	6.00 (6.00,8.00)	8.00 (6.00,10.00)	−7.562	<0.001

1 Categorical variables are presented as counts (%), and between-group comparisons were made using the χ² test.

2 Continuous variables are presented as M (P25, P75), and the Mann–Whitney *U* test was used.

A two-sided *P* value of less than 0.05 was considered statistically significant.

WBC, White blood cell count; NLR, Neutrophil-to-lymphocyte ratio; CRP, C-reactive protein; PLT, Platelet; PLR, Platelet-to-lymphocyte ratio; MPV, Mean platelet volume; PCT, Procalcitonin; SF, Serum ferritin; IL-6, Interleukin 6; PT, Prothrombin time; APTT, Activated partial thromboplastin time.

### 3.3 Baseline characteristics of the training and test cohorts

A total of 1,573 children were randomly assigned to the training set (*n* = 1,104) and internal validation set (*n* = 469) in a 7:3 ratio. There were no significant differences in baseline characteristics between the two cohorts (*P* > 0.05), indicating that they were well balanced and comparable, as shown in Table 2.

**Table 2:** Baseline characteristics of patients in the training set and internal validation set.

Variables	Training set (*n* = 1,104)	Internal validation set (*n* = 469)	*χ*^2^/Z	*P*
Calendar month of onset, *n* (%)^1^			10.739	0.465
January, *n* (%)^1^	254 (23.01)	112 (23.88)		
February, *n* (%)^1^	200 (18.12)	78 (16.63)		
March, *n* (%)^1^	71 (6.43)	36 (7.68)		
April, *n* (%)^1^	263 (23.82)	102 (21.75)		
May, *n* (%)^1^	148 (13.41)	55 (11.73)		
June, *n* (%)^1^	21 (1.90)	11 (2.35)		
July, *n* (%)^1^	20 (1.81)	6 (1.28)		
August, *n* (%)^1^	13 (1.18)	8 (1.71)		
September, *n* (%)^1^	5 (0.45)	2 (0.43)		
October, *n* (%)^1^	5 (0.45)	7 (1.49)		
November, *n* (%)^1^	32 (2.90)	20 (4.26)		
December, *n* (%)^1^	72 (6.52)	32 (6.82)		
Male, *n* (%)^1^	671 (60.78)	276 (58.85)	0.512	0.474
Prematurity, *n* (%)^1^	53 (4.80)	22 (4.69)	0.009	0.925
Allergy history, *n* (%)^1^	103 (9.33)	47 (10.02)	0.183	0.669
Wheezing History, *n* (%)^1^	85 (7.70)	36 (7.68)	0.000	0.987
Cyanosis, *n* (%)^1^	20 (1.81)	6 (1.28)	0.574	0.449
Fever, *n* (%)^1^	728 (65.94)	316 (67.38)	0.304	0.581
Cough, *n* (%)^1^	1,091 (98.82)	460 (98.08)	1.312	0.252
Wheezing, *n* (%)^1^	265 (24.00)	107 (22.81)	0.258	0.612
Dyspnea, *n* (%)^1^	66 (5.98)	28 (5.97)	0.000	0.995
Lung consolidation, *n* (%)^1^	62 (5.62)	25 (5.33)	0.051	0.821
Pleural Fluid, *n* (%)^1^	13 (1.18)	10 (2.13)	2.082	0.149
Pneumothorax, *n* (%)^1^	1 (0.09)	0 (0.00)	0.425	0.514
Breastfeeding, *n* (%)^1^	607 (54.98)	263 (56.08)	0.160	0.689
Admitted to the PICU, *n* (%)^1^	78 (7.07)	32 (6.82)	0.030	0.863
Admission weight (kg), M (Q₁, Q₃)^2^	10.35 (8.00,14.00)	10.00 (8.00,14.00)	−0.750	0.453
Age (months), M (Q₁, Q₃)^2^	13.00 (5.66,31.00)	12.00 (5.75,30.00)	−0.449	0.653
RSV-CLASS, M (Q₁, Q₃)^2^	2.00 (2.00,3.00)	2.00 (2.00,3.00)	−0.040	0.968
WBC (×10^9^/L), M (Q₁, Q₃)^2^	8.82 (6.53,11.66)	9.06 (6.82,11.04)	−0.161	0.872
NLR, M (Q₁, Q₃)^2^	0.55 (0.28,1.14)	0.51 (0.26,1.11)	−0.786	0.432
CRP (mg/L), M (Q₁, Q₃)^2^	2.01 (0.50,7.97)	2.36 (0.50,7.85)	−0.623	0.533
PLT (×10^9^/L), M (Q₁, Q₃)^2^	356.00 (280.25,432.00)	356.00 (282.00,434.50)	−0.428	0.668
PLR, M (Q₁, Q₃)^2^	68.89 (45.26,109.83)	69.67 (45.67,106.57)	−0.132	0.895
MPV (fL), M (Q₁, Q₃)^2^	9.00 (8.50,9.80)	9.00 (8.50,9.80)	−0.853	0.394
PCT (ng/mL), M (Q₁, Q₃)^2^	0.10 (0.06,0.15)	0.10 (0.06,0.17)	−1.087	0.277
SF (ng/mL), M (Q₁, Q₃)^2^	61.73 (29.36,109.30)	67.84 (32.10,109.75)	−1.396	0.163
IL-6 (pg/mL), M (Q₁, Q₃)^2^	8.60 (3.00,21.10)	9.01 (3.00,20.08)	−0.419	0.676
PT (s), M (Q₁, Q₃)^2^	11.50 (10.80,12.50)	11.50 (10.80,12.49)	−1.002	0.317
APTT (s)	30.09 (26.80,33.20)	30.01 (26.91,33.20)	−0.132	0.895
D-Dimer (ug/mL), M (Q₁, Q₃)^2^	0.33 (0.23,0.46)	0.33 (0.22,0.48)	−0.615	0.538
AT-III (%), M (Q₁, Q₃)^2^	103.95 (94.50,111.10)	103.40 (94.55,110.50)	−0.672	0.501

1 Categorical variables are presented as counts (%), and between-group comparisons were made using the χ² test.

2 Continuous variables are presented as M (P25, P75), and the Mann–Whitney *U* test was used.

A two-sided *P* value of less than 0.05 was considered statistically significant.

WBC, White blood cell count; NLR, Neutrophil-to-lymphocyte ratio; CRP, C-reactive protein; PLT, Platelet; PLR, Platelet-to-lymphocyte ratio; MPV, Mean platelet volume; PCT, Procalcitonin; SF, Serum ferritin; IL-6, Interleukin 6; PT, Prothrombin time; APTT, Activated partial thromboplastin time.

### 3.4 Univariable analysis of risk factors for secondary bacterial infection with *S. pneumoniae* and/or *H. influenzae*

In the training set of 1,104 patients, 140 developed co-infection with *S. pneumoniae* and/or *H. influenzae*, corresponding to an infection rate of 12.68%. Univariate analysis revealed that the co-infection group had significantly higher proportions or levels of prematurity, cyanosis, fever, wheezing, dyspnea, lung consolidation, pleural fluid, pneumothorax, non-breastfeeding, admitted to the PICU, RSV-CLASS, WBC, NLR, CRP, PLR, PCT, SF, IL-6, PT, APTT, and D-dimer compared with the non-bacterial infection group (all *P* < 0.05), suggesting that these factors might be potential influencing variables, as shown in Table 3.

**Table 3:** Univariate analysis of risk factors for secondary bacterial infection with *S. pneumoniae* and/or *H. influenzae* in the training cohort.

Variables	No Bacterial Coinfection (*n* = 964)	Bacterial Coinfection (*n* = 140)	*χ*^2^/Z	*P*
Calendar month of onset, *n* (%)^1^			9.924	0.537
January, *n* (%)^1^	220 (22.82)	34 (24.29)		
February, *n* (%)^1^	173 (17.95)	27 (19.29)		
March, *n* (%)^1^	67 (6.95)	4 (2.86)		
April, *n* (%)^1^	228 (23.65)	35 (25)		
May, *n* (%)^1^	133 (13.8)	15 (10.71)		
June, *n* (%)^1^	16 (1.66)	5 (3.57)		
July, *n* (%)^1^	18 (1.87)	2 (1.43)		
August, *n* (%)^1^	10 (1.04)	3 (2.14)		
September, *n* (%)^1^	5 (0.52)	0 (0)		
October, *n* (%)^1^	5 (0.52)	0 (0)		
November, *n* (%)^1^	27 (2.8)	5 (3.57)		
December, *n* (%)^1^	62 (6.43)	10 (7.14)		
Male, *n* (%)^1^	577 (59.85)	94 (67.14)	2.724	0.099
Prematurity, *n* (%)^1^	39 (4.05)	14 (10.00)	9.483	0.002
Allergy history, *n* (%)^1^	92 (9.54)	11 (7.86)	0.411	0.521
Wheezing History, *n* (%)^1^	71 (7.37)	14 (10.00)	1.194	0.274
Cyanosis, *n* (%)^1^	8 (0.83)	12 (8.57)	41.188	<0.001
Fever, *n* (%)^1^	625 (64.83)	103 (73.57)	4.155	0.042
Cough, *n* (%)^1^	953 (98.86)	138 (98.57)	0.087	0.768
Wheezing, *n* (%)^1^	214 (22.20)	51 (36.43)	13.569	<0.001
Dyspnea, *n* (%)^1^	34 (3.53)	32 (22.86)	81.265	<0.001
Lung consolidation, *n* (%)^1^	28 (2.9)	34 (24.29)	105.433	<0.001
Pleural Fluid, *n* (%)^1^	8 (0.83)	5 (3.57)	7.896	0.005
Pneumothorax, *n* (%)^1^	0 (0.00)	1 (0.71)	6.892	0.009
non-breastfeeding, *n* (%)^1^	420 (43.57)	77 (55.00)	6.454	0.011
Admitted to the PICU, *n* (%)^1^	42 (4.36)	36 (25.71)	84.924	<0.001
Admission weight (kg), M (Q₁, Q₃)^2^	10.50 (8.03,14.00)	10.00 (7.70,13.00)	−1.310	0.190
Age (months), M (Q₁, Q₃)^2^	13.00 (5.69,31.00)	12.00 (5.43,30.00)	−0.780	0.435
RSV-CLASS, M (Q₁, Q₃)^2^	2.00 (2.00,3.00)	2.00 (2.00,3.00)	−4.835	<0.001
WBC (×10^9^/L), M (Q₁, Q₃)^2^	8.57 (6.36,11.24)	10.45 (7.71,13.95)	−5.545	<0.001
NLR, M (Q₁, Q₃)^2^	0.48 (0.26,0.97)	1.29 (0.70,2.52)	−10.194	<0.001
CRP (mg/L), M (Q₁, Q₃)^2^	1.52 (0.44,5.70)	13.04 (4.33,25.39)	−11.693	<0.001
PLT (×10^9^/L), M (Q₁, Q₃)^2^	351.00 (281.00,428.75)	372.00 (272.50,485.25)	−1.863	0.062
PLR, M (Q₁, Q₃)^2^	65.77 (43.60,103.38)	98.55 (65.88,156.12)	−6.094	<0.001
MPV (fL), M (Q₁, Q₃)^2^	9.00 (8.40,9.78)	9.20 (8.63,9.80)	−1.454	0.146
PCT (ng/mL), M (Q₁, Q₃)^2^	0.10 (0.06,0.13)	0.32 (0.14,0.65)	−13.216	<0.001
SF (ng/mL), M (Q₁, Q₃)^2^	57.98 (27.38,104.73)	95.17 (52.55,169.40)	−6.384	<0.001
IL-6 (pg/mL), M (Q₁, Q₃)^2^	6.85 (2.70,15.61)	39.63 (22.87,65.97)	−13.123	<0.001
PT (s), M (Q₁, Q₃)^2^	11.40 (10.70,12.44)	11.90 (11.31,12.70)	−4.343	<0.001
APTT (s), M (Q₁, Q₃)^2^	29.91 (26.70,32.80)	30.96 (28.03,35.48)	−2.669	0.008
D-Dimer (ug/mL), M (Q₁, Q₃)^2^	0.33 (0.22,0.45)	0.40 (0.30,0.54)	−4.641	<0.001
AT-III (%), M (Q₁, Q₃)^2^	104.05 (94.50,111.50)	103.60 (93.88,110.20)	−1.318	0.187

1 Categorical variables are presented as counts (%), and between-group comparisons were made using the χ² test.

2 Continuous variables are presented as M (P25, P75), and the Mann–Whitney *U* test was used.

A two-sided *P* value of less than 0.05 was considered statistically significant.

WBC, White blood cell count; NLR, Neutrophil-to-lymphocyte ratio; CRP, C-reactive proteinl; PLT, Platelet; PLR, Platelet-to-lymphocyte ratio; MPV, Mean platelet volume; PCT, Procalcitonin; SF, Serum ferritin; IL-6, Interleukin 6; PT, Prothrombin time; APTT, Activated partial thromboplastin time.

### 3.5 Development of the predictive model via LASSO regression

All independent variables, except for CRP, PCT, and dyspnea, were entered into the LASSO regression. The optimal *λ* value was selected via ten-fold cross-validation (Figure 2). At the optimal penalty coefficient *λ* = 0.027032, the coefficients of cyanosis, lung consolidation, RSV-CLASS, WBC, NLR, SF, and IL-6 were not shrunk to zero, suggesting that these variables might be influencing factors for co-infection with *S. pneumoniae* and/or *H. influenzae*. The changes in coefficients of all variables as *λ* varies are presented in Figure 3.

**Figure 2:**
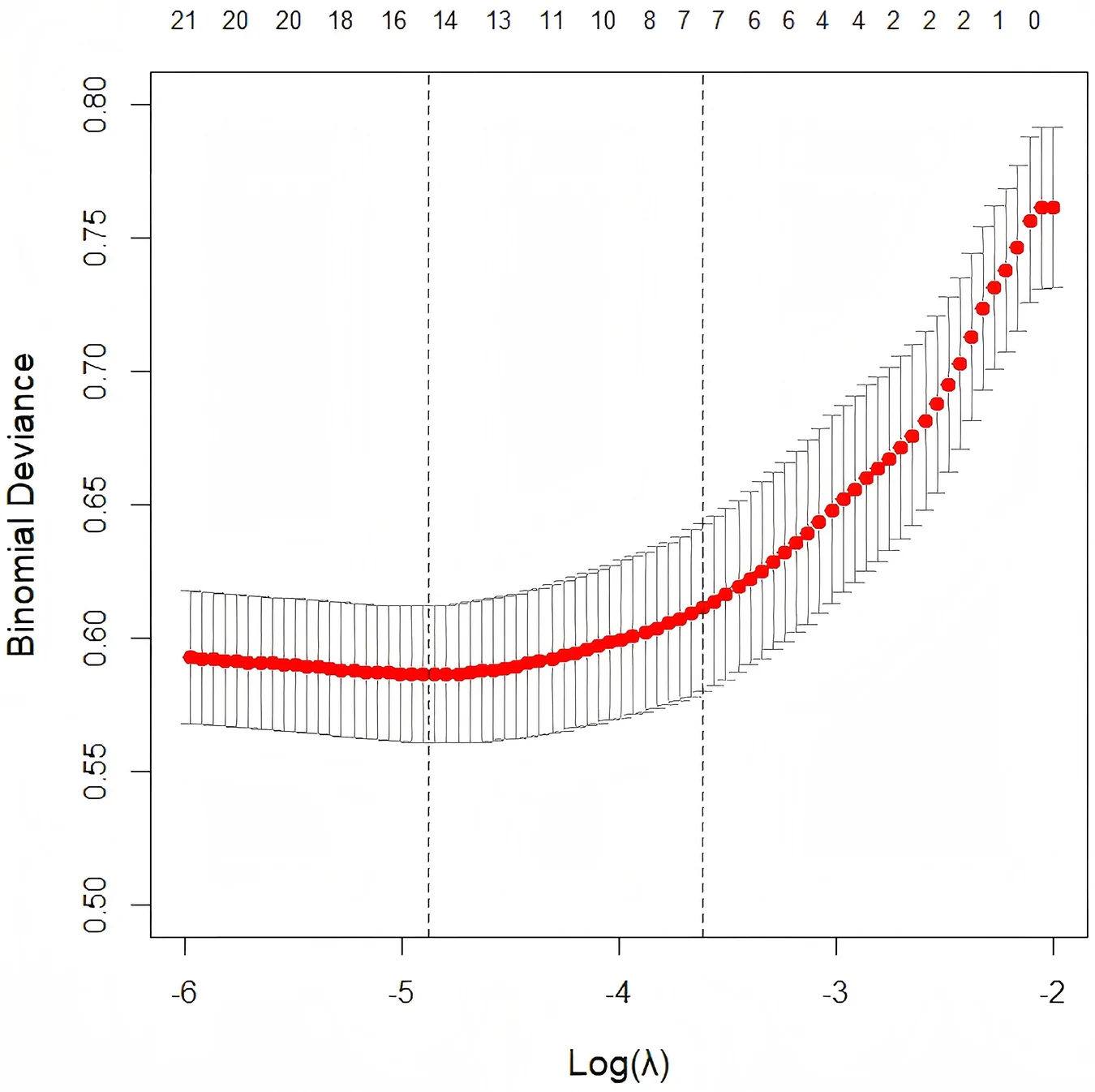
Diagram of 10-fold cross-validation.

**Figure 3:**
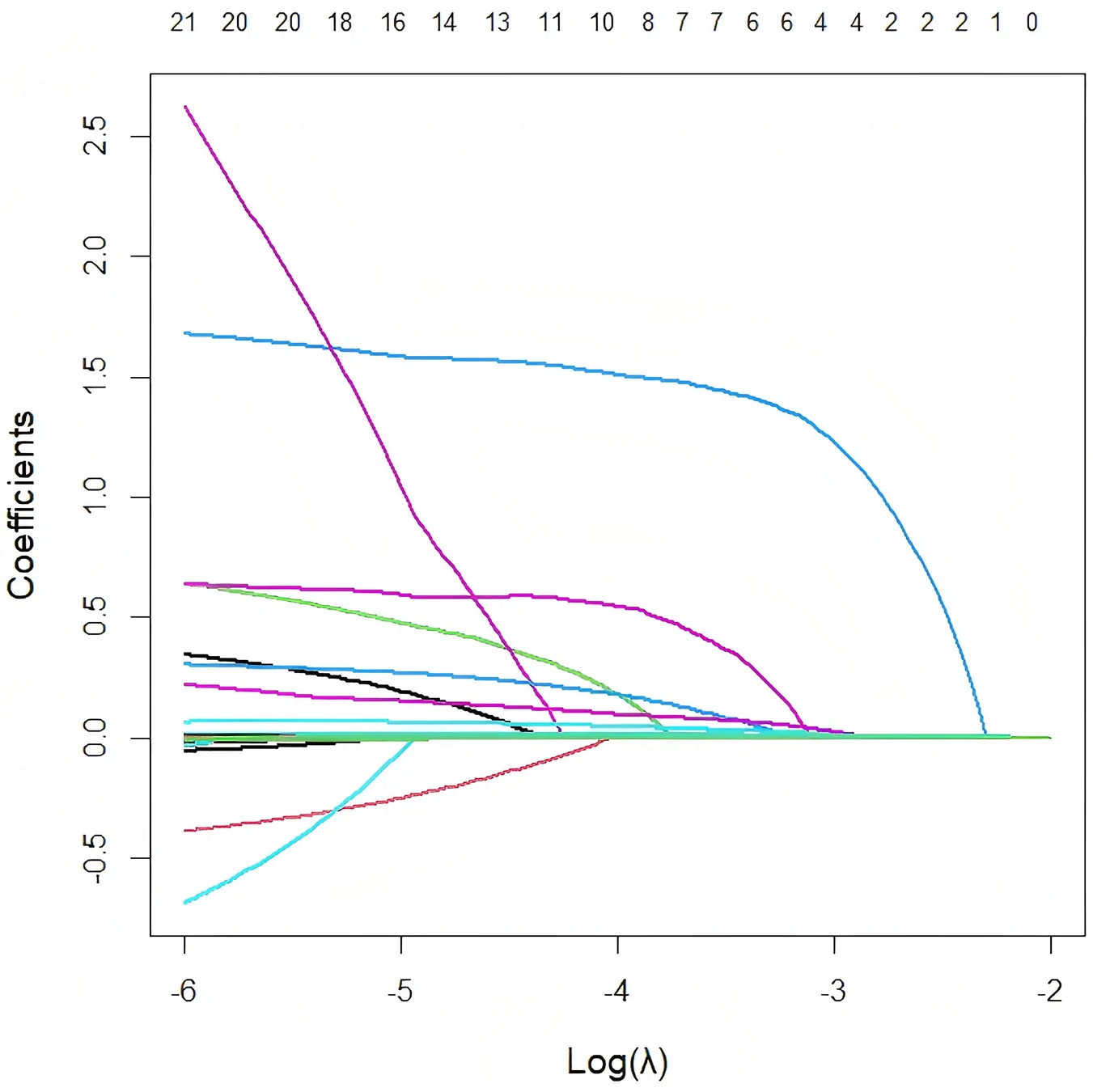
Convergence path of LASSO regression coefficients.

### 3.6 Multivariable logistic regression analysis of secondary bacterial infection in the training set

A binary logistic regression analysis was performed with the presence of co-infection with *S. pneumoniae* and/or *H. influenzae* as the dependent variable and the seven predictors selected by LASSO regression as independent variables. The results showed that lung consolidation, RSV-CLASS, WBC, NLR, SF, and IL-6 were independent influencing factors for co-infection, as shown in Table 4.

**Table 4:** Multivariate logistic regression analysis of secondary bacterial infection with *S. pneumoniae* and/or *H. influenzae* in the training cohort.

Variables	*β*	SE	OR (95% CI)	*p*-Value
Cyanosis	0.996	0.591	2.707 (0.85–8.62)	0.092
Lung consolidation	1.714	0.33	5.551 (2.91–10.588)	<0.001
RSV-CLASS	0.376	0.128	1.456 (1.134–1.871)	0.003
WBC	0.083	0.023	1.087 (1.04–1.136)	<0.001
NLR	0.142	0.054	1.153 (1.037–1.282)	0.009
SF	0.002	0.001	1.002 (1.001–1.004)	0.005
IL-6	0.017	0.003	1.017 (1.012–1.022)	<0.001
Intercept	−4.785	0.417		<0.001

Intercept represents the baseline log-odds of co-infection when all independent predictors in the final model are zero.

WBC, White blood cell count; NLR, Neutrophil-to-lymphocyte ratio; SF, Serum ferritin; IL-6 Interleukin 6.

### 3.7 Nomogram for clinical application

Based on the results of the logistic regression analysis, we selected lung consolidation, RSV-CLASS, WBC, NLR, SF, and IL-6 as independent predictors and constructed a nomogram model for predicting the risk of co-infection with *S. pneumoniae* and/or *H. influenzae*. The probability of co-infection for an individual patient can be estimated by summing the scores assigned to each predictor (Figure 4).

**Figure 4:**
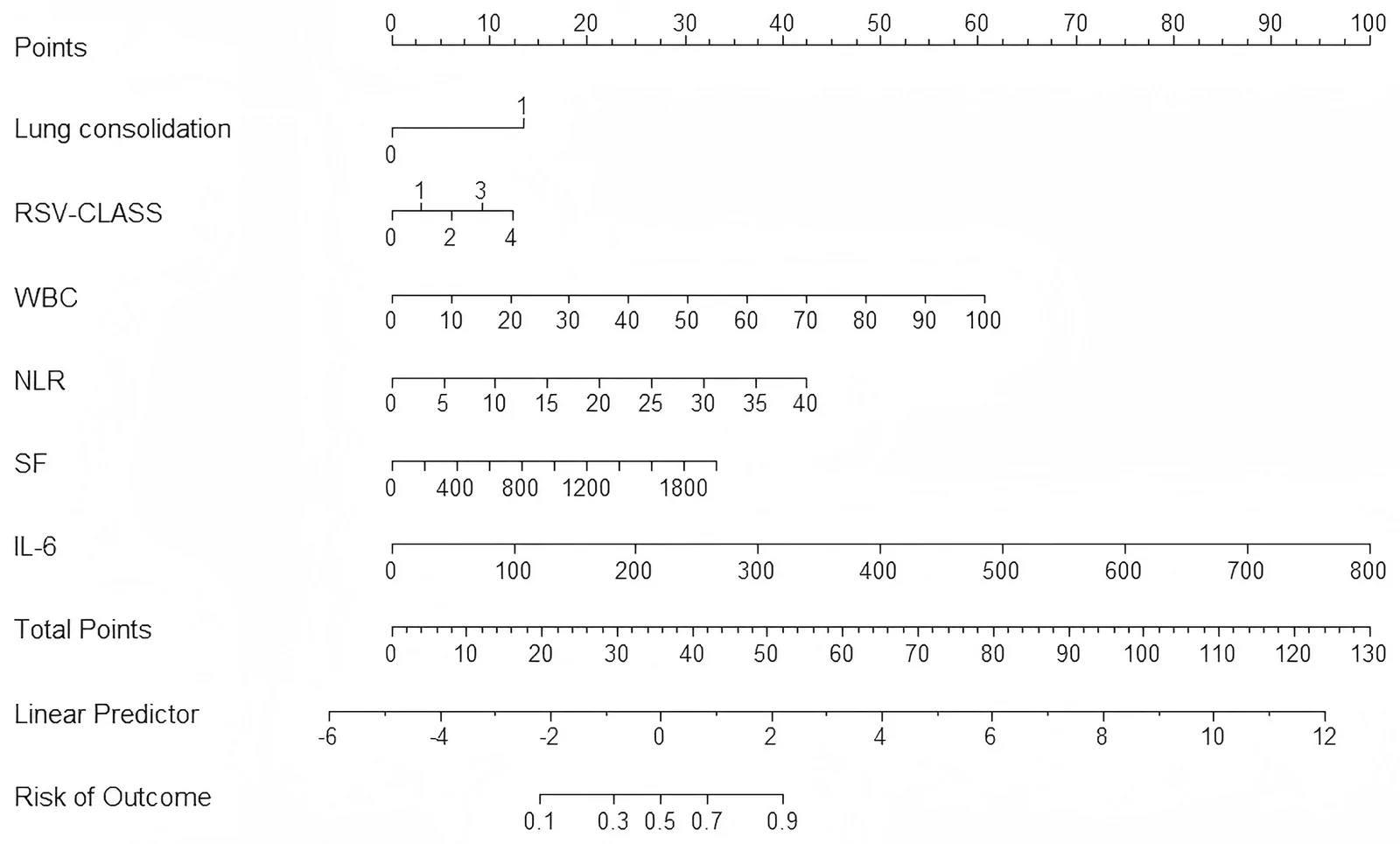
The nomogram for predicting secondary bacterial infection with *S. pneumoniae* and/or *H. influenzae*.

### 3.8 Performance and validation of the predictive model

Receiver operating characteristic (ROC) curves were plotted for both the training and test sets. For the training set, the AUC was 0.872 (95% CI: 0.841–0.898), with an optimal cut-off value of 0.109, yielding a sensitivity of 0.757, a specificity of 0.814, and a Youden index of 0.571. At this cut-off, with a prevalence of 12.7%, the positive predictive value (PPV) was approximately 0.37 and the negative predictive value (NPV) was approximately 0.96. The optimism-corrected C-statistic derived from Bootstrap resampling was 0.864, and the shrinkage factor was 0.9726, indicating a low risk of overfitting. In the test set, the AUC was 0.829 (95% CI: 0.780–0.877) (Figure 5), confirming that the model had good discriminative ability and generalizability. In an exploratory analysis applying the model to the full bacteria-positive cohort (*n* = 498, including training-set patients), the AUC was 0.850 (95% CI: 0.817–0.883). However, as this cohort overlapped with the training set, this estimate is apparent rather than independently validated (Supplementary Figure S1). To provide an independent out-of-sample estimate, we further applied the model to the bacteria-positive subgroup restricted to the internal validation set only (*n* = 151, none of whom were used in model development). In this independent subgroup, the AUC was 0.823 (95% CI: 0.757–0.889) (Supplementary Figure S2).

**Figure 5:**
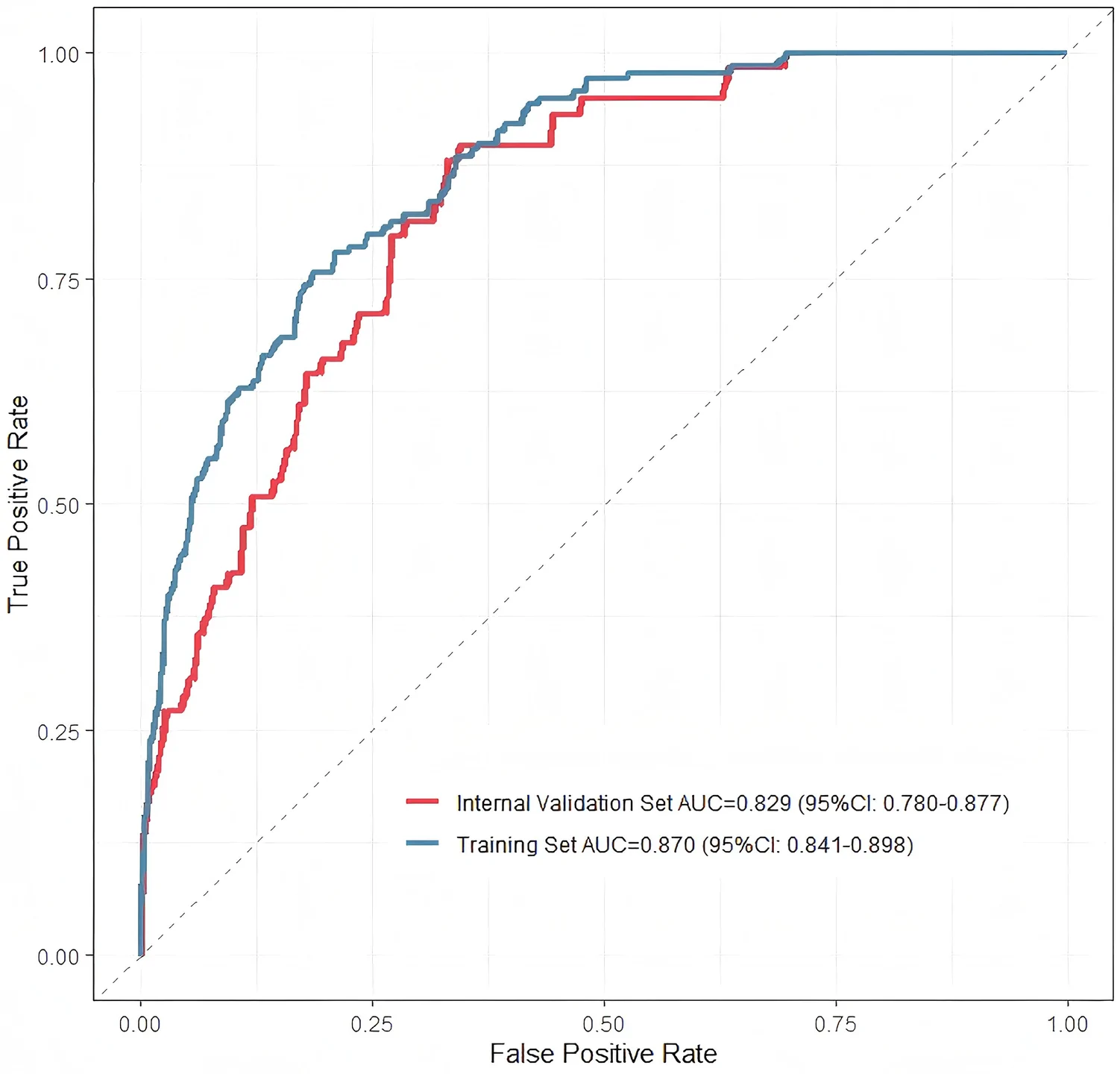
ROC curves: discriminative performance of the nomogram in the training and internal validation sets.

After Bootstrap correction, the calibration intercept and slope in the training set were −0.1229 and 0.9312, respectively, indicating good overall calibration. In the test set, the calibration intercept and slope were −0.350 and 0.762, respectively, and the Hosmer–Lemeshow test yielded a P-value > 0.05, indicating that the overall calibration performance was within a clinically miscalibration (Figures 6, 7). Decision curve analysis (Figures 8, 9) demonstrated that, in both the training and test sets, the net benefit of model-guided decisions exceeded that of the “treat-all” and “treat-none” strategies within the threshold probability range of 0.2 to 0.6. Notably, the Youden-derived cut-off of 0.109 lies below this DCA-favourable threshold range. Therefore, if the clinical goal is specifically to reduce unnecessary antibiotic use, a higher decision threshold (0.2–0.6) may be more appropriate, as it would improve PPV despite some loss in sensitivity. This suggests acceptable clinical utility within this threshold range, although the calibration results indicate overfitting and highlight the need for external validation to confirm generalizability.

**Figure 6:**
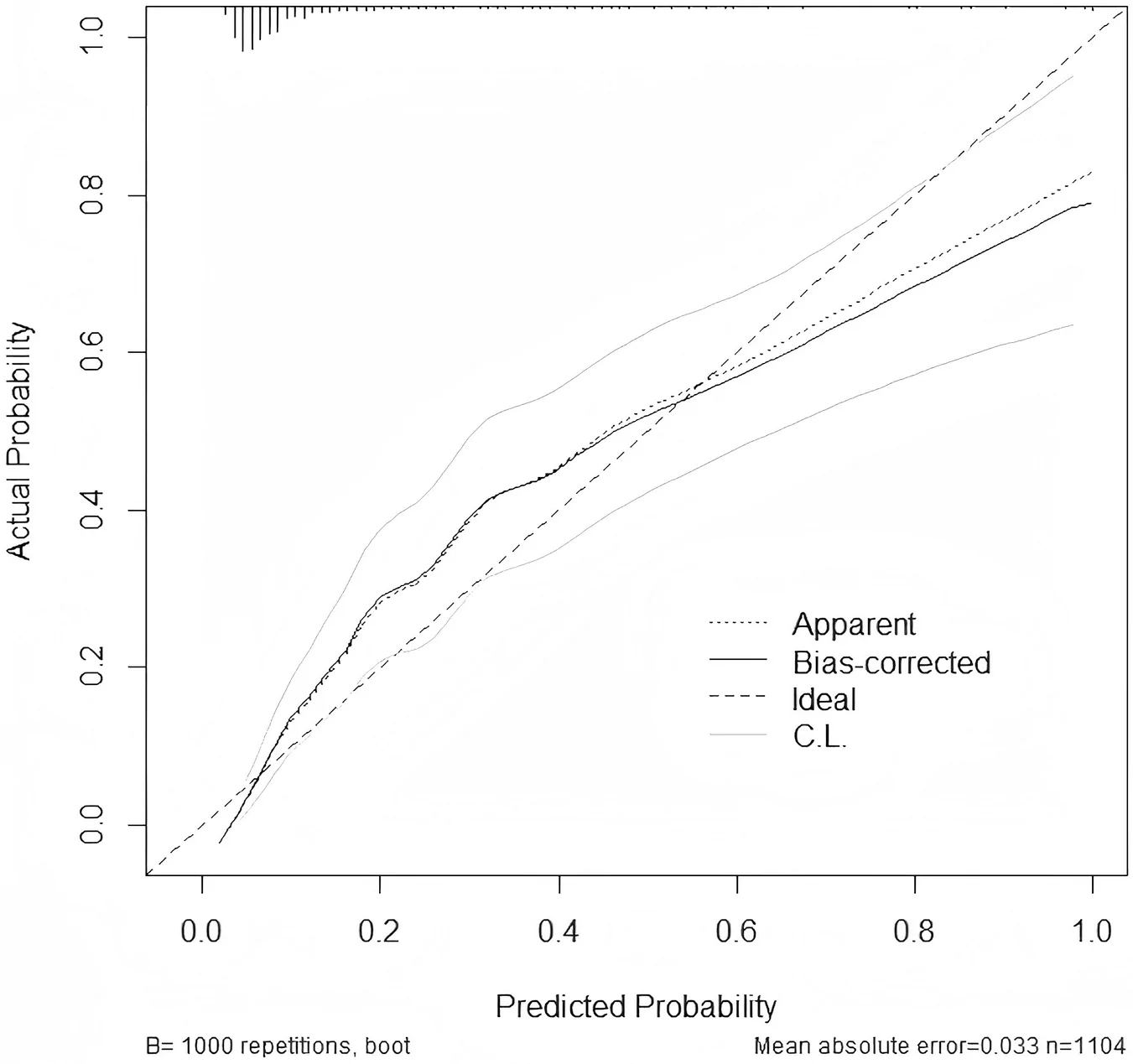
Calibration plot of the training set, where the *y*-axis represents the observed probability and the *x*-axis represents the predicted probability; the dashed diagonal line represents ideal prediction.

**Figure 7:**
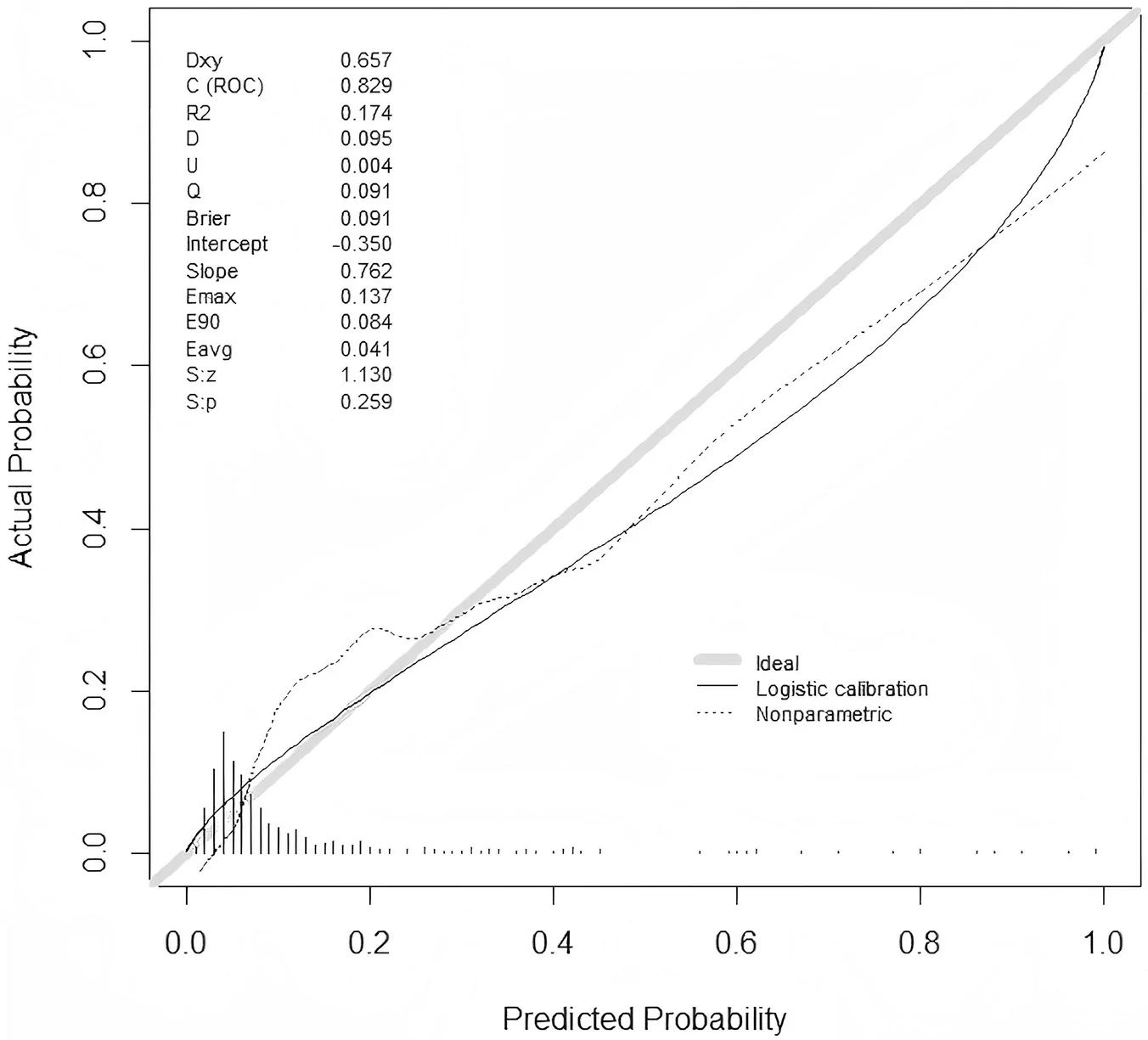
Calibration assessment in the internal validation set.

**Figure 8:**
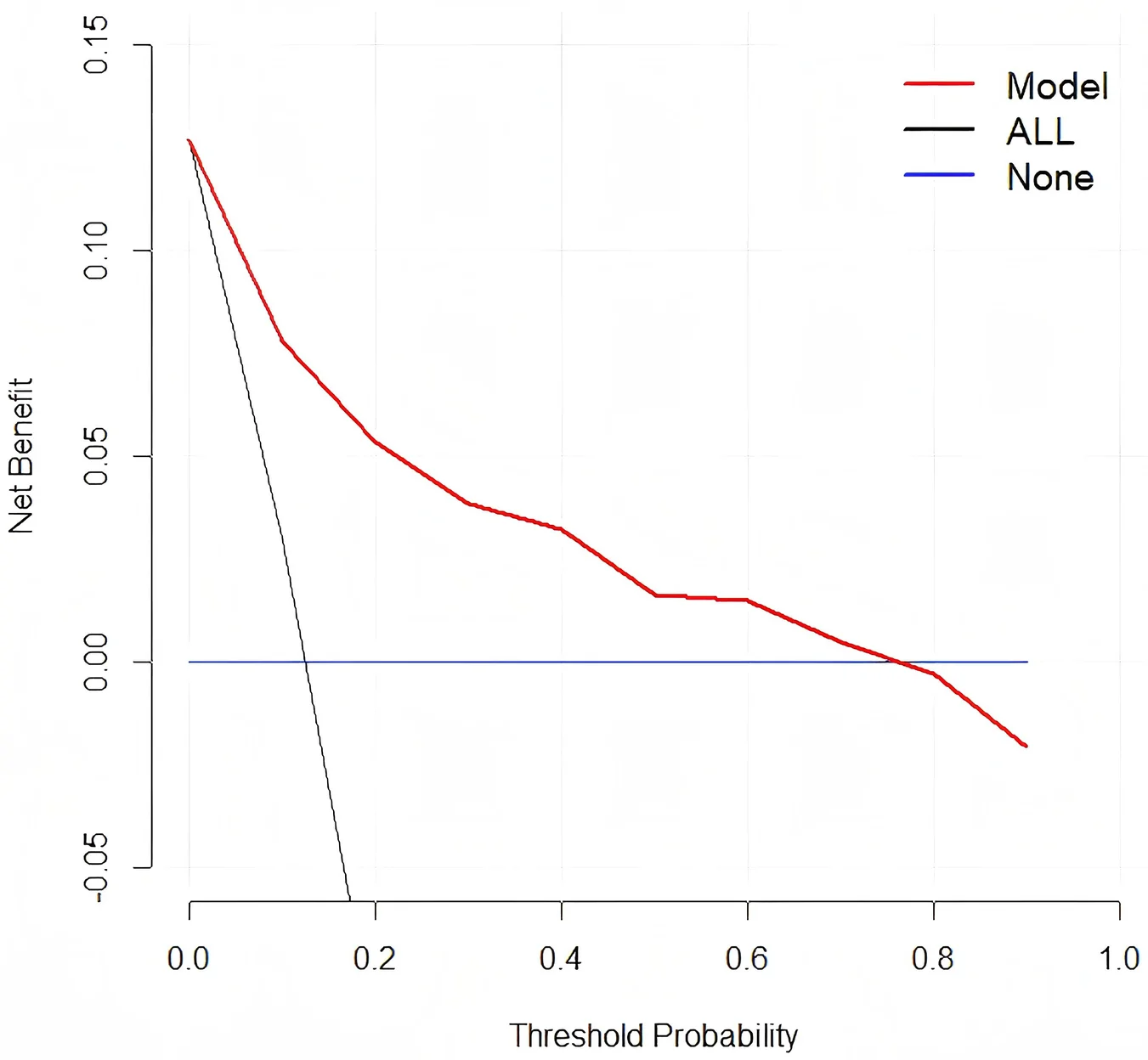
Clinical utility assessment by decision curve analysis (DCA) in the training set, with the *y*-axis representing net benefit. The red solid line represents the nomogram. The blue line represents the “treat-none” strategy, and the black horizontal line represents the “treat-all” strategy.

**Figure 9:**
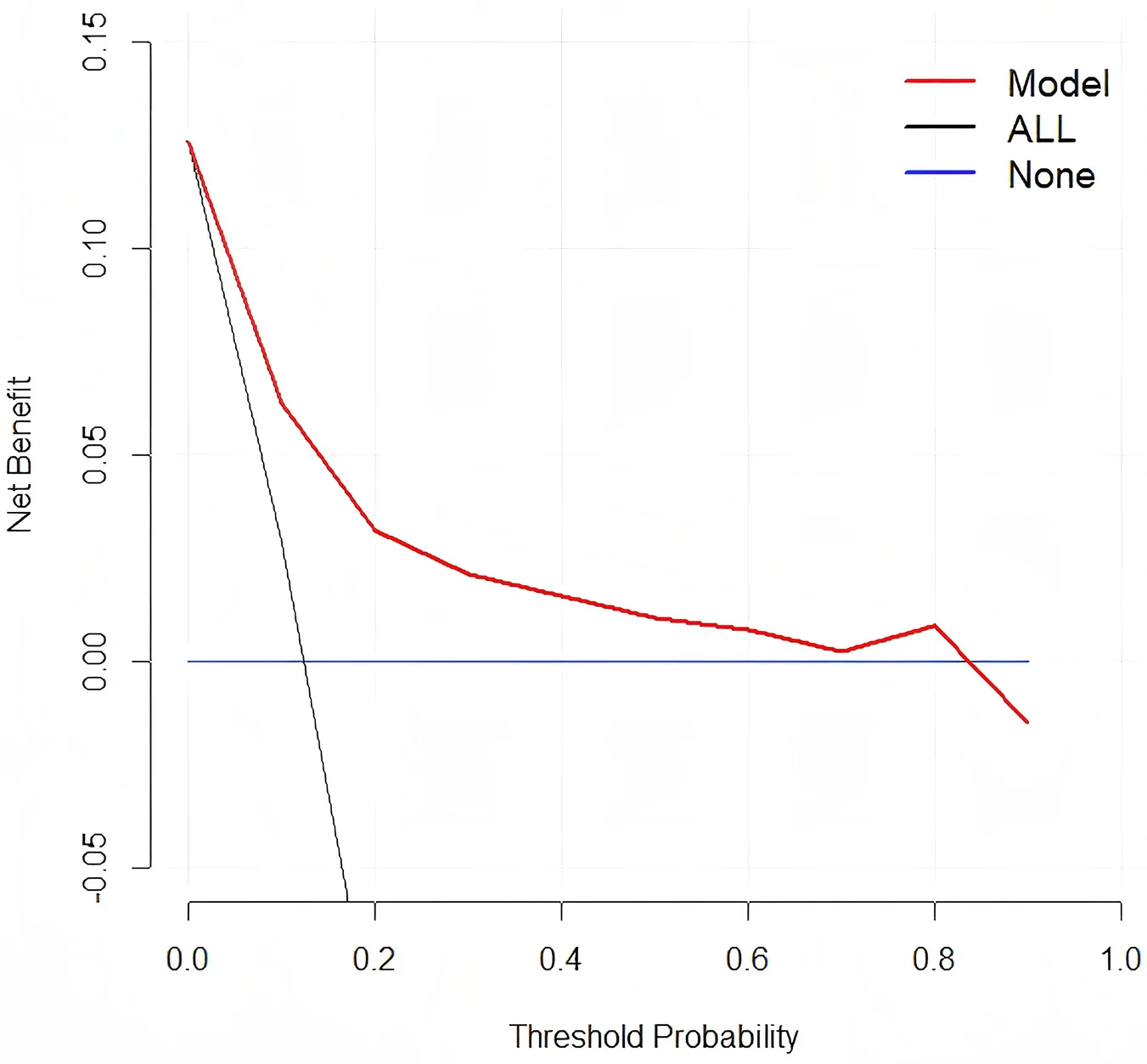
Decision curve analysis (DCA) of the nomogram in the internal validation set.

## 4 Discussion

We included 1,573 children hospitalised with RSV infection, 498 (31.66%) of whom tested positive for *S. pneumoniae* and/or *H. influenzae*. However, only 199 (12.65%) met our predefined criteria for true bacterial co-infection. To help clinicians decide whether to start antibiotics, we developed and internally validated a nomogram to predict such co-infection in this population. The final model included six predictors, lung consolidation, RSV-CLASS, WBC, NLR, SF, and IL-6, covering imaging, clinical symptoms and laboratory tests. In the training set (*n* = 1,104), the infection rate was 12.68% (140 events), with an AUC of 0.872 (95% CI: 0.841–0.898). In the internal validation set, the AUC was 0.829 (95% CI: 0.780–0.877), and the optimism-corrected C-statistic was 0.864.

### 4.1 Imaging

Our study showed that lung consolidation carried the greatest weight in the final model (OR: 5.551, 95% CI: 2.910–10.588). Previous studies have demonstrated that RSV co-infection with bacteria is closely associated with specific clinical symptoms, radiological changes, biomarkers, and disease severity (13). In a study of 128 infants with RSV infection, children with bacterial co-infection more frequently presented with lobar or segmental consolidation (20). When RSV infects ciliated epithelial cells, it impairs mucociliary clearance, allowing bacteria colonising the nasopharynx to reach the lower respiratory tract. In addition, RSV disrupts the airway and alveolar epithelial barriers, enhancing bacterial replication and predisposing patients to severe bacterial pneumonia (21). Therefore, in clinical practice, when a chest radiograph of a child with RSV infection reveals consolidation, clinicians should be vigilant for secondary *S. pneumoniae* and/or *H. influenzae* infection and maintain a low threshold for timely antibiotic administration.

### 4.2 Clinical symptoms

RSV-CLASS is a severity scoring system derived from a prospective study of more than 700 RSV-infected children over six winter seasons (2014–2020). It comprises four items: cough, tachypnoea, rales, and wheezing, each scored as 1 point. A higher total score indicates more severe clinical manifestations and a greater likelihood of developing lower respiratory tract infection (18). In our study, RSV-CLASS was identified as an independent predictor of co-infection with *S. pneumoniae* and/or *H. influenzae* (OR: 1.456, 95% CI: 1.134–1.871). These signs reflect the degree of airway inflammation and mucus obstruction; when secondary bacterial invasion occurs, the additional increase in secretions and mucosal oedema tends to make these clinical signs more pronounced. Previous studies have also shown that RSV-infected children with nasopharyngeal carriage of *S. pneumoniae* and *H. influenzae* exhibit more severe clinical presentations, higher rates of hospital admission, and longer hospital stays (4). From a pathophysiological perspective, RSV infection can disrupt the airway and alveolar epithelial barrier, creating conditions conducive to secondary bacterial infection. Previous experimental studies have shown that prior RSV infection can enhance bacterial replication, rendering the host more susceptible to developing severe bacterial pneumonia (21). In the setting of secondary bacterial infection, not only is the inflammatory response within the lung parenchyma directly aggravated, but the synergistic interaction between the virus and bacteria further amplifies the inflammatory cascade. This leads to a marked deterioration in both ventilation and gas exchange, clinically manifesting as severe respiratory distress signs such as cyanosis and dyspnea (21).

### 4.3 Laboratory tests

In our study, WBC, NLR, SF, and IL-6 were independent predictors of co-infection with *S. pneumoniae* and/or *H. influenzae* in children hospitalised with RSV, and these four markers reflect the inflammatory response from different angles. WBC has long been the most readily available inflammatory marker in paediatric practice, but its ability to separate bacterial from viral infection is limited, mainly because viral infections themselves often raise the white cell count. Even so, it remained an independent predictor here (OR: 1.087, 95% CI: 1.040–1.136). This is in line with Li et al. (22), who reported that WBC, CRP and PCT were all higher in children with bacterial respiratory infection than in those with viral or Mycoplasma infection, and that combining them improved diagnostic accuracy. Elevated NLR reflects an immune state characterised by neutrophil activation and lymphocyte suppression, which is closely related to the stress response during bacterial infection (14). In our study, the odds ratio for NLR was 1.153 (95% CI: 1.037–1.282). This finding is consistent with numerous studies in sepsis and intra-abdominal infections, where NLR has been proven to be a crucial indicator for predicting infection severity and prognosis (23). SF is an acute-phase protein. Its transcription is increased by inflammatory cytokines such as TNF-α, IL-1β and IL-6 (24), and the protein also participates in iron sequestration, a mechanism of nutritional immunity by which the host restricts iron availability to bacteria (24). Using a cutoff of 295.5 ng/mL, Gehlot et al. reported 86% specificity and 99% negative predictive value for blood-culture-positive sepsis (24). SF therefore seems better suited to ruling out bacterial infection than to confirming it. IL-6 acts further upstream in this response. It is released early by macrophages, monocytes and endothelial cells after bacterial stimulation and drives hepatic CRP synthesis (25). Slaats et al. (25) described the IL-6/CRP programme as characteristic of bacterial infection, in contrast to the IL-18/ferritin pattern seen in viral infection. Our previous study also found IL-6 to be an independent predictor of *S. pneumoniae* and/or *H. influenzae* co-detection in children with RSV (15).

Our study addressed the critical issue of distinguishing colonizers from true pathogens. Previous studies have commonly used positive bacterial detection as the grouping criterion. However, existing research shows that the bacterial co-infection rate in children with RSV infection ranges from 15.7% to 39% (7, 9–12), a wide variation. Moreover, the nasopharyngeal carriage rate of *S. pneumoniae* in children can reach 36.4–42.1%, and that of *H. influenzae* can reach 26.7–36.5% (26). Given such a high bacterial detection rate, relying solely on microbiological evidence—without stringent clinical and laboratory indicators of infection—would easily lead to misclassifying colonizers as true pathogens, resulting in an overestimation of infection risk by the model and misguided antibiotic decisions. Therefore, we constructed a multi-dimensional assessment incorporating bacteriological findings, clinical manifestations, and elevated inflammatory markers to rigorously define infection, thereby making our prediction model methodologically more robust. This model better reflects real-world clinical decision-making—in practice, the question is not whether bacteria are present in the respiratory tract, but whether they require antibiotic treatment.

The limitations of this study include the following. First, the retrospective design did not incorporate bacterial load or serum amyloid A (SAA) (16), limiting assessment of bacterial burden on infection determination. Second, the composite reference standard itself carries a risk of classification bias. Some children may have only upper respiratory tract bacterial colonization but present with significant inflammatory responses due to other causes, leading to their misclassification as bacterial infections. Conversely, a small number of children with true invasive bacterial infection may have their clinical manifestations or inflammatory markers still below peak levels at the time of specimen collection, failing to accurately reflect the true inflammatory state *in vivo*, and may thus be misclassified as having no bacterial infection. Therefore, future model development could consider incorporating dynamic, continuously monitored indicators to improve predictive performance. Third, to avoid reduced statistical power from insufficient sample size, *S. pneumoniae* and *H. influenzae* were combined into a single group, and independent predictors for each bacterium were not explored separately. Fourth, the calibration drop from training to validation (slope 0.762) suggests notable overfitting, likely due to the retrospective single-center design and limited events. At the Youden cut-off of 0.109, the PPV was only ≈0.37, meaning most children flagged as “positive” would not have infection. We therefore propose using the model primarily as a rule-out aid (NPV ≈ 0.96) rather than a standalone indication for antibiotics. Raising the threshold to 0.2–0.6 would improve PPV but reduce sensitivity. Additionally, antibiotic utilisation data were unavailable, so prospective multi-centre validation is required before clinical use. Fifth, detection methods varied across the cohort, and allocation tracked severity—intubated children more often received mNGS, which is more sensitive than culture or RT-PCR. That's a bias, and it favours our hypothesis. Future studies should standardise testing across severity groups to avoid this. Sixth, this was a single-centre study. The patient mix, clinical practices, and testing protocols at our institution may not reflect those in other settings, so the model may not travel well to other populations or healthcare systems. External validation in diverse, multi-centre cohorts is essential before the model can be recommended for broader use.

We recognize that many pediatric centers do not routinely test IL-6 or ferritin in children with RSV, so our model may not generalize directly to those settings. At our hospital, these markers were available for nearly all patients because of our institutional protocol, but elsewhere clinicians would have to order them separately—which could introduce selection pressure or limit practical use. To improve real-world applicability, future work should test whether a simpler model, using only widely available variables (e.g., vital signs, oxygen saturation, and routine blood counts without inflammatory markers), performs similarly well. Until that is done, we recommend restricting our current model to centers that routinely measure these parameters, or interpreting its results with caution when they are not.

## 5 Conclusions

This study developed and internally validated a nomogram for predicting secondary infection with *S. pneumoniae* and/or *H. influenzae* in hospitalised children with RSV. The model incorporates six independent predictors—lung consolidation, RSV-CLASS, WBC, NLR, SF and IL-6. While the model demonstrated good performance in our centre, calibration in the validation set was suboptimal (slope 0.762), and at our chosen cut-off, the PPV was only about 0.37. That means the model's real strength lies in identifying low-risk children who can safely avoid antibiotics, not in telling us who definitely needs them. Its value for broader clinical implementation requires confirmation through rigorous external validation.

## Data Availability

The raw data supporting the conclusions of this article will be made available by the authors, without undue reservation.
