# Supplementary material for: Predictors of secondary streptococcus pneumoniae and/or haemophilus influenzae infection in children hospitalised with RSV, and development of a clinical prediction model to inform antibiotic decisions: a retrospective cohort study based on coloniser–pathogen distinction
Source: Front Pediatr. 2026 Oct 28;14:1915356. doi: 10.3389/fped.2026.1915356 (PMC13751928; doi:10.3389/fped.2026.1915356)
Supplement: Supplementary file 1 [file Supplementaryfile1.docx]

Supplementary Table S1. Missing data.

| Variable | Missing, n | Missing, % |
| --- | --- | --- |
| Prematurity | 6 | 0.38 |
| Allergy history | 1 | 0.06 |
| Wheezing History | 1 | 0.06 |
| Cyanosis | 2 | 0.13 |
| Breastfeeding | 25 | 1.59 |
| WBC | 2 | 0.13 |
| NLR | 1 | 0.06 |
| CRP | 1 | 0.06 |
| PLT | 1 | 0.06 |
| PLR | 3 | 0.19 |
| MPV | 33 | 2.1 |
| PCT | 24 | 1.53 |
| SF | 177 | 11.25 |
| IL-6 | 18 | 1.14 |
| PF | 139 | 8.84 |
| APTT | 138 | 8.77 |
| D-dimer | 140 | 8.9 |
| AT-III | 256 | 16.27 |

The number of imputations was set to 5, and all candidate predictors and the outcome were included in the imputation model.

The original nomogram overpredicted risk in the validation set (calibration slope = 0.762, intercept = –0.350). To obtain recalibrated estimates, apply the following formula:

LP_corrected = (LP_original - Intercept_cal) / Slope_cal = (LP_original + 0.350) / 0.762.

Supplementary Table S2. TRIPOD+AI statement

| **#** | **TRIPOD Item** | **Corresponding section in manuscript** | **Content/Status** |
| --- | --- | --- | --- |
| 1 | Title | Title | Revised to: “Predictors of secondary *Streptococcus pneumoniae* and/or *Haemophilus influenzae* infection in children hospitalised with RSV, and development and internal validation of a clinical prediction model to inform antibiotic decisions: a retrospective cohort study based on coloniser–pathogen distinction” |
| 2 | Abstract | Abstract | Structured abstract; include training set (n=1104) and internal validation set (n=469); 6 predictors; training AUC 0.872, bootstrap-corrected C-statistic 0.864; validation AUC 0.829; conclusion states internally validated. |
| 3 | Background and rationale | Introduction | Completed. |
| 4 | Objectives | Introduction (final paragraph) | Revised to: “To develop and internally validate a nomogram for predicting secondary S. pneumoniae and/or H. influenzae infection…” |
| 5 | Source of data | Methods (Study Design) | Single-centre retrospective cohort, Jan 2023–Oct 2025, Luoyang Maternal and Child Health Hospital. |
| 6 | Participants | Methods + Figure 1 | Completed: 1,573 children included; Figure 1 shows participant flow. |
| 7 | Outcome | Methods (Variables) | Composite definition described. Add: “Outcome status was determined before any statistical analysis based on the predefined composite reference standard. The outcome assessors were not involved in the model development process.” |
| 8 | Predictors | Methods (Variables) + Tables 1/2 | Completed. Add: “Outcome status was determined before any statistical analysis based on the predefined composite reference standard. The outcome assessors were not involved in the model development process.” |
| 9 | Sample size | Methods (Statistical Analysis) | Training set n=1104, outcome events n=140; final model includes 6 predictors; EPV = 140/6 ≈ 23.33, exceeding the recommended ≥10. |
| 10 | Missing data | Methods + Supplementary | Multiple imputation used; details (imputed variables, number of imputations, diagnostics) in Supplementary Material. |
| 11 | Statistical analysis methods | Methods (Statistical Analysis) | LASSO with 10-fold cross-validation; multivariable logistic regression; internal validation with 1,000 bootstrap resamples repeating full modelling strategy; optimism-corrected C-statistic; uniform shrinkage factor 0.9726. |
| 12 | Development vs. validation | Methods + Results | Model developed only in training set; internal validation set used solely for performance evaluation. |
| 13 | Model specification | Results (Nomogram) + Figure 4 | Nomogram includes 6 predictors: lung consolidation, RSV-CLASS, WBC, NLR, SF, IL-6. |
| 14 | Model performance | Methods + Results | Report AUC, bootstrap-corrected C-statistic, calibration (intercept, slope, Hosmer–Lemeshow), and DCA. |
| 15 | Results: Participants | Results + Table 1 + Figure 1 | Completed: 1,573 children; 199 outcome events (12.65%). |
| 16 | Results: Model development | Results (LASSO + Logistic) | Training set n=1104, events n=140; LASSO selected 7 variables; final multivariable model retained 6 independent predictors. |
| 17 | Results: Model specification | Results + Figure 4 | Nomogram displayed; full regression equation with shrinkage-corrected coefficients in Supplementary Material. |
| 18 | Results: Model performance | Results + Figures 5–9 | Training AUC 0.872 (95% CI 0.841–0.898); bootstrap-corrected C-statistic 0.864; shrinkage factor 0.9726. Validation AUC 0.829 (95% CI 0.780–0.877). Bacterial-positive subgroup AUC 0.850 (95% CI 0.817–0.883). Calibration intercept/slope: training −0.1229/0.9312; validation −0.350/0.762; HL P>0.05. DCA net benefit over threshold probabilities 0.2–0.6. |
| 19 | Limitations | Discussion | Add: only internal validation performed; independent external validation required before clinical use. |
| 20 | Interpretation | Discussion | Completed. |
| 21 | Implications | Discussion + Conclusions | Completed. |
| 22 | Supplementary information and funding | Funding + Supplementary | State TRIPOD checklist and multiple imputation details in Supplementary Material; funder had no role in study design, data collection, analysis, or manuscript preparation. |

Supplementary Figure S1. Subgroup diagnostic performance of the prediction model in the bacterial‑positive subgroup (colonisation + infection group, n = 498).

The model achieved an AUC of 0.850 (95% CI 0.817–0.883) in this subgroup, indicating that it maintained satisfactory discriminatory performance in identifying true bacterial infection among patients with detected bacteria.

Supplementary Figure S2. ROC curve of the nomogram in the bacteria‑positive subgroup of the validation set

The model was applied to 151 children with bacterial detection (59 true infections and 92 colonisations) who were not used in model development. The area under the curve (AUC) was 0.823 (95% CI: 0.757–0.889), indicating good discriminatory ability in distinguishing colonisation from true infection among bacteriologically positive children. The diagonal dashed line represents the reference of no discrimination (AUC = 0.5). Shaded area denotes the 95% confidence band.
